# Impact of Collected and Recycled Concrete Plant Washing Water on the Physical, Chemical, and Mechanical Properties of Mortars

**DOI:** 10.3390/ma18071641

**Published:** 2025-04-03

**Authors:** Bechara Haddad, Farjallah Alassaad, Nassim Sebaibi

**Affiliations:** 1 Builders Lab, Builders Ecole d’ingénieurs, COMUE Normandie Université, 1 Rue Pierre et Marie Curie, 14610 Epron, France; nassim.sebaibi@builders-ingenieurs.fr; 2 CMEG, Z.A. de Cardonville, Rue Compagnie D, 14740 Thue et Mue, France; farjallah.alassaad@gmail.com

**Keywords:** recycled wash water, collected wash water, mortar properties, sustainability, mechanical performance, environmental impact

## Abstract

The management of washing water from concrete plants is a growing environmental and industrial concern due to its high alkalinity and the presence of suspended solids, chemical admixtures, and dissolved salts. This study investigates the impact of collected and recycled concrete plant washing water on the physical, chemical, and mechanical properties of mortars. Two types of wastewater were analyzed: (1) collected water (CW), obtained from settling tanks with residual suspended particles and chemical compounds, and (2) recycled water (RW), subjected to a complete treatment process including pH stabilization and solid particle removal. The effects of these waters were compared against potable water (PW) through a comprehensive experimental program evaluating the porosity, density, shrinkage, and mechanical performance of mortars. The results indicate that using CW and RW leads to increased porosity, higher shrinkage, and a reduction in compressive and flexural strength, with RW having a more pronounced impact. These changes are attributed to the chemical composition of the water, which affects cement hydration and matrix densification. Despite these drawbacks, the proper treatment and controlled usage of such waters may offer sustainable alternatives to potable water in concrete production, contributing to resource conservation and environmental sustainability.

## 1. Introduction

The construction sector has undergone a period of constant evolution, marked by the introduction of novel methodologies that prioritize efficiency, feasibility, and reduced construction times [1,2,3,4,5,6,7].

Ready-mix concrete plants play an essential role in the development of modern infrastructures, contributing to the boom in urban and industrial construction. However, their operation generates by-products, notably wash water, which poses complex environmental challenges. This water, generated by the cleaning of production equipment and mould trucks, is often heavily contaminated with fine particles from cementitious materials, oils, chemical admixtures, and alkalis. As a result, these waters have a high alkalinity (pH > 11) and a significant concentration of suspended solids, making them unsuitable for direct discharge without prior treatment [8,9].

This issue is becoming increasingly important in a global context marked by the scarcity of freshwater resources. Today, some 3.6 billion people live in water-stressed areas, and this figure could rise to 5 billion by 2050 [10]. The concrete industry, which consumes around 1.6% of the world’s freshwater resources, plays an important role in this pressure [11]. For example, producing one cubic meter of concrete requires around 150 L of water [12], while cleaning activities for truck-mixers and production equipment generate hundreds of liters of wastewater per day and per site. As an indication, some concrete plants can consume up to 10,000 L of water per day just for cleaning, amplifying the overall environmental impact of this industry [8].

To meet these challenges, concrete plants use advanced treatment systems to purify and stabilize wash water. The process begins with settling in successive basins, eliminating the coarsest solid particles and reducing the pollutant load. These loads are the surplus from the plant or from the concrete mixers washed by the water. This means gravel, sand, cement, and supplementary cementing materials. The next phase consists of separating oils and hydrocarbons, using specific devices to ensure that the water is free of immiscible substances that could alter the properties of the materials. Oils, fuels, and other chemicals come from the oil in the equipment, as well as from the admixtures in the concrete. Finally, a pH adjustment is carried out by injecting carbon dioxide (CO_2_), for example, which neutralizes alkalinity and stabilizes the chemical composition of the water [8,12]. As we know, concrete is an alkaline medium, so washing it makes the water alkaline. After this treatment, this water can be discharged, but we do not know whether it can be reused in the manufacture of concrete.

In addition to these conventional steps, some plants are innovating by integrating advanced technologies, such as ultrafiltration or electrocoagulation, to further improve the quality of the recycled water. These approaches make it possible to treat finer contaminants and guarantee optimum compatibility with modern concrete formulations [12]. Numerous studies show that recycled water treated in this way can be used safely while offering significant environmental benefits, such as a reduced carbon footprint and better management of natural resources.

The use of recycled wash water in concrete and mortar formulations has grown in popularity over the last few decades thanks to its potential to meet the construction industry’s demands for sustainability and efficiency. Pioneering studies, such as those by Sandrolini and Franzoni (2001), have demonstrated that the use of recycled water, even with high alkalinity, can maintain mechanical strength in excess of 90% compared to references using potable water [8]. In addition, this practice reduces capillary porosity thanks to the filling effect of fine suspended particles. More recently, Aruntaş et al. (2022) confirmed that treated wastewater can be used to produce class C30/37 concrete with mechanical performance close to standard while significantly reducing the water footprint of production [12].

However, raw wash water contains fine cement particles, concrete residues, and chemical additives that can alter the consistency, setting, and mechanical strength of mixes. To overcome these effects, advanced treatment techniques have been developed. For example, the electrocoagulation studied by Matos et al. (2020) guarantees constant water quality, even for complex modern concrete formulations [11]. Another method, carbonation, involves injecting carbon dioxide into the wash water to stabilize suspended solids and limit their chemical activity. This approach improves concrete properties, increasing compressive strength and reducing negative effects on workability while contributing to CO_2_ sequestration [13].

The mechanical performance of concretes made with treated wash water is often equivalent or even superior to that of conventional concretes. The use of this water reduces capillary porosity and improves durability, helping to extend the service life of concrete structures [8]. These results show that recycled water, if properly treated, can meet modern performance requirements. The addition of water must not affect the strength required for the safe handling of prefabricated panels [14,15,16,17,18].

In addition to the mechanical advantages, recycling wash water in concrete batching plants significantly reduces environmental impact. By reducing the release of drinking water and untreated discharges into the environment, this practice is in line with the principles of sustainability. What is more, stabilized solids from the washing water can be reused as partial cement substitutes, reducing the overall carbon footprint of concrete. One study estimated that this process reduces carbon impact by around 14% compared with traditional methods [19]. In addition, innovative bioleaching techniques that involve the utilization of volatile fatty acids generated during the process of dark fermentation have emerged as a viable solution for the effective management of concrete waste from construction and demolition operations [20]. This methodology, examined within the framework of moving-bed reactors, provides a sustainable approach for the recovery and reuse of valuable resources within the construction sector, thereby contributing to a more circular management of materials. These ecological benefits go hand in hand with reduced costs, making this approach economically attractive.

To ensure widespread adoption of this practice, it is essential to standardize processes and define precise quality thresholds for recycled water. Technologies such as CO_2_ stabilization [21,22,23] and filtration need to be integrated into modern concrete plant operations. Large-scale research confirms that these practices can be implemented efficiently while complying with industrial requirements. This standardization would not only improve the durability and performance of concretes but would also support a transition to a more environmentally friendly construction industry.

Studies converge on the idea that recycled wash water, when properly treated, can be used in partial or total substitution of potable water in concrete formulations [8,11,19,21,24,25,26,27,28]. However, final concrete performance is highly dependent on the quality of the treatment applied and the chemical composition of the recycled water. This research provides a solid framework for exploring the possibilities of integrating these waters into industrial practices while identifying the limits and precautions to be respected to guarantee optimum performance.

The aim of this study is to examine in detail the influence of two types of wash water on mortar properties. The first type, collected water, comes from settling tanks and still contains suspended particles and chemical residues, representing an intermediate stage in the treatment process. The second type, recycled water, is the result of a complete treatment process incorporating chemical stabilization and solid particle reduction, making it suitable for reuse in concrete formulations.

The specific objectives are to examine the physical properties of mortars made from these waters, particularly in terms of density, porosity, and consistency. They also aim to analyze the mechanical performance of the mortars, with particular emphasis on compressive and flexural strength. Finally, these results will be compared with those obtained using standard drinking water as a reference in order to identify the limits and opportunities associated with the use of such water in an industrial setting.

Ultimately, this study seeks to provide practical recommendations for the effective integration of recycled water into mortar manufacturing processes while guaranteeing performance in line with the requirements of modern worksites. This approach is part of a broader vision of sustainable development aimed at reducing dependence on natural resources while limiting the environmental impact of the construction industry.

## 2. Materials and Methods

The following section provides a detailed description of the materials and methods used in this study.

### 2.1. Cement

Portland cement type CEM II/A-LL 52.5R, known for its rapid hardening and high strength, is the binder used in this study. It is commonly used in building construction where high mechanical performance is required. The cement’s chemical composition and main mechanical properties, including compressive strengths at different hardening ages, are shown in Table 1.

### 2.2. Sand

In this study, natural washed sand conforming to fine aggregate standards was used. The granulometric distribution complies with normative requirements for fine aggregates. The sand is clean, impurity-free, and has a specific gravity of 2.63. Its characteristics include density, water absorption, and other relevant properties. Table 2 shows the main properties of the sand for use in mortar and concrete formulations.

### 2.3. Water

In this research, an in-depth and rigorously structured study was carried out on the types of water used in mortar formulations. These waters come from a concrete mixing plant specializing in the production of admixture-enriched concrete. Three types of water were compared: two from the plant’s industrial processes and a reference drinking water. The aim was to gain a better understanding of their characteristics and assess their influence on the physico-chemical and mechanical properties of concrete.

Water treatment is a multifaceted process involving a series of sequential stages. Initially, cascade sedimentation of coarse particles is employed, followed by an oil and hydrocarbon separation process. Subsequently, pH stabilization is achieved by injecting CO_2_, which facilitates partial alkalinity neutralization.

The first type of water analyzed is referred to as collected water (CW), coming from the second settling tank. This water undergoes an intermediate treatment stage where coarse particles, such as gravel and sand, are removed. However, fine particles, chemical contaminants (chlorides, sulfates), and dissolved organic matter remain. These compounds can interact with the concrete’s constituents, altering its mechanical and durability properties.

**Figure 1:**
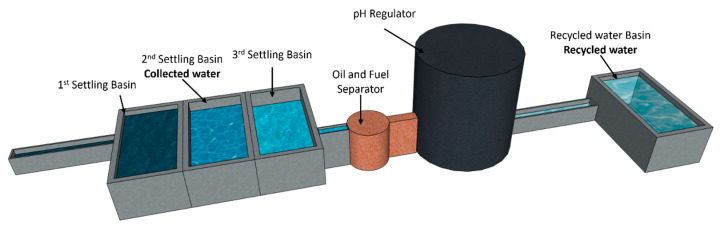
Wash water treatment system.

The second type is recycled water (RW), the result of a complete industrial recycling process. This process includes three settling basins to remove solid particles, an oil and hydrocarbon separator, and pH adjustment by injection of carbon dioxide (CO_2_). This treatment stabilizes the water’s physico-chemical properties and guarantees its compliance with industrial standards for concrete. This system was implemented because the collected water cannot be discharged into the sumps without prior treatment due to its chemical composition and the associated environmental risks. The treatment system is illustrated in Figure 1.

Finally, a reference potable water complying with the strict standards of NF EN 1008 [29] for mixing water is used to provide a standard basis for comparison. This comparative methodology is essential to systematically assess the advantages and limitations of recycled water.

To characterize these waters, a number of physical and chemical tests were carried out. The results obtained are summarized in Table 3 below.

The analyses reveal marked differences between the three types of water. The collected water, although cleaned of coarse particles, still has high concentrations of suspended solids (3400 mg/L), chlorides, and sulfates, as well as a high alkaline pH (10.9 at 20 °C). These characteristics could compromise the durability and resistance of mortars. Recycled water, on the other hand, thanks to its advanced treatment, has properties comparable to those of drinking water, particularly in terms of pH, suspended solids, and mineral ion content.

### 2.4. Spread Mortar Test

The mortar is meticulously prepared according to a precise formulation, ensuring a standard water-to-binder ratio is adhered to. Following homogenization, the mixture is meticulously poured into a truncated cone-shaped mold positioned centrally on the impact table. Filling is methodically executed in a single layer, avoiding excessive compaction, and the surface is subsequently smoothed with a spatula. The mold is then removed vertically and without lateral movement, enabling the mortar to spread freely. The table is then operated to make 15 successive drops in 15 s, with each drop measuring 10 mm in height. Upon completion of the test, the largest and smallest diameters of the spread mortar are measured and averaged. This result is then used to assess the consistency of the mortar in accordance with standard NF EN 1015-3 [30]: the higher the spread, the more fluid the mortar.

### 2.5. Porosity and Density

In accordance with standard NF P18-459 [31], water-accessible porosity and density of mortar samples were tested. Widely referred to as the “vacuum water test”, this method determines both the sample’s open porosity and bulk density. Specimens are initially dried to a constant mass before being placed in a desiccator connected to a vacuum pump. A 24 h high vacuum is applied to extract the air contained in the open pores of the samples. This is followed by the filling of the desiccator with demineralized water, allowing complete impregnation of the pores under vacuum for a further 24 h. When re-saturation is complete, hydrostatic weighing is used to measure the immersed mass of the samples. Samples are then superficially wiped with a damp cloth to obtain their saturated mass. These mass measurements are combined to give the water-accessible porosity and apparent density according to the formulas given in standard NF P18-459.

### 2.6. Water Absorption by Immersion

The water absorption of mortars was determined by using a simple direct immersion protocol. Samples of hardened mortar were first oven-dried to constant mass to remove residual water. Their dry mass was then carefully recorded. Mortar is considered dry when the variation in mass between two successive measurements taken 24 h apart does not exceed 0.1%. Samples were then fully immersed in demineralized water for a period of 24 h to allow total saturation by capillary absorption. The utilization of demineralized water is predicated on its chemical neutrality, thereby ensuring that no interaction occurs with the cement’s hydrated phases. After this period of immersion, the samples were taken out of the water, superficially wiped with a damp cloth, and their saturated mass was measured. Water absorption rate as a percent was calculated to be an increase in the sample mass due to the absorption of water in relation to the initial sample dry mass.

### 2.7. Flexural Strength

The mortar flexural strength test was carried out by a three-point bending process according to the French standard NF EN 196-1 [32]. Samples of each mortar formulation showed prismatic forms of around 40 × 40 × 160 mm^3^. After the curing, these specimens are submitted to a three-point bending test, by which a specimen is merely laid on two lower supports and loaded at the middle by a third upper support that goes down with constant speed until the failure occurs. The force exerted by the maximal load at the moment of the flexure failure is recorded. The flexural strength is then calculated according to the formula prescribed by the standard, depending on the breaking load, the span between lower supports, and the cross-sectional dimensions of the specimen.

### 2.8. Compressive Strength

After the three-point bending tests, the two prismatic halves obtained from each test were subjected to a compression test according to NF EN 196-1 [32]. The compression machine’s platen can receive a test dimension of 40 mm × 40 mm × 40 mm. An axial compressive load was increased at a constant rate of 2.4 kN/s according to the standard. For both, the compressive strength, Rc, was measured by using the maximum record of force during the instance of crushing failure for each of these specimens by means of a standardized formula that also includes the dimensions of their cross-section. In the present test, which represents the complement of the previous bending test, the mechanical properties of the different mortars developed are fully characterized.

### 2.9. Total Shrinkage

The total shrinkage of the different mortar formulations was measured, for which the French standard NF P 15-433 [33] sets out the method for measuring dimensional variations on hardened mortar specimens. Prismatic specimens measuring 40 × 40 × 160 mm^3^ were cast in molds and demolded after 24 h; these specimens were fitted at their ends with metal pins, which enabled accurate measurement of the length variations. The specimens, after demolding, were stored in an air-conditioned room at 50% relative humidity and 20 °C for curing under controlled conditions.

The dimensional variations in the specimens were measured during different time intervals by means of a high-precision length comparator. Then, total shrinkage was calculated using the ratio of length variation to initial specimen length.

### 2.10. Formulation

In this work, two sets of formulation series were performed to investigate the comparative performance of the blends using the different types of water. These formulations have been evaluated against potable water-based formulations as a standard reference.

First series of formulations: potable water was gradually substituted with collected water. Second series of formulations: a progressive substitution of potable water with recycled water was carried out. In substitution rates, the rates of the two formulation series were set at 25%, 50%, 75%, and 100%.

These experiments have been carried out in such a way that the resulting formulations’ physical and mechanical properties can be compared to those obtained with tap water. Table 4 summarizes all the formulations studied, which shows the proportions of substitution of water and the nature of the water used. It should be noted that mortars made with collected water were more fluid than other formulations. This is due to the presence of admixtures, notably superplasticizers, which come from concrete production. To quantify this effect, a spread test was performed on each formulation.

## 3. Results

### 3.1. Spread Mortar Test

**Figure 2:**
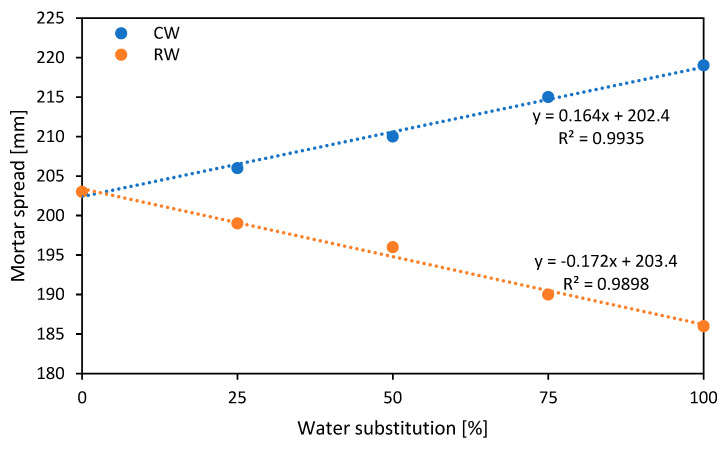
Spread mortar test results.

Results in Figure 2 show that the addition of CW leads to a progressive increase in the spread of the mortar, from 203 mm for the reference mortar to 219 mm with total replacement of potable water. This trend is explained by the possible presence of superplasticizer residues in the recycled water, which improves the flowability of the mixture [26]. On the other hand, the use of RW progressively reduces the spread, reaching 186 mm for 100% recycled water. This suggests that the impurities and fine particles present in the recycled water alter the rheology of the mortar, limiting its workability [26]. So, while collected water can improve workability, it needs to be controlled to avoid excessive flowability, while recycled water can make the mortar stiffer, affecting its application and workability.

### 3.2. Porosity and Density

The results obtained in Table 5 show a progressive increase in porosity and a decrease in mortar density as drinking water is replaced by collected water (CW) or recycled water (RW). Porosity rises from 19% for the reference to 24% for RW100, reflecting a more porous internal structure. At the same time, the density decreases, reaching 1990 kg/m^3^ for RW100 compared to 2010 kg/m^3^ for the reference. These trends show that the use of water from industrial processes has a significant impact on the microstructure of the mortar, with potential implications for mechanical performance and durability.

One of the main causes of this increase in porosity could be the increased presence of suspended solids and fine particles in collected and recycled water. These particles, often inert, disrupt the compactness of the cementitious matrix by acting as non-reactive inclusions. They thus limit the continuity of cement hydration products, such as hydrated calcium silicate (C-S-H), which is essential for mortar cohesion and density [34,35,36].

Additionally, the chemical composition of these waters, particularly their sulfate and chloride content, exerts a significant influence on cement hydration and the microstructure of mortars. Sulfates have been observed to promote the formation of expansive ettringite, resulting in altered microstructure and increased porosity [37]. Similarly, sulfate attack has been shown to induce the degradation of cement hydration products, leading to a reduction in density and a weakening of the cementitious network [38,39]. Furthermore, the distribution of C-S-H gels is modified by the chlorides present, altering cement cohesion and accelerating its structural deterioration [40].

These phenomena may explain both the increase in porosity and the decrease in density observed in formulations with a higher proportion of non-potable water.

### 3.3. Total Shrinkage

The results in Table 6 show a significant increase in shrinkage, particularly at 28 days, when potable water is replaced by collected or recycled water in mortar formulations. Shrinkage at 28 days increases from 715 µm/m for the reference to 740 µm/m for CW100 and 815 µm/m for RW100. This progressive increase is particularly marked in formulations using a higher proportion of recycled water, suggesting a direct influence of the physico-chemical characteristics of this water on mortar behavior.

One of the main explanations for this trend is linked to the increased content of dissolved salts, notably chlorides and sulfates, that are present in collected and recycled water. These salts modify the chemical composition of the cement’s liquid phase and can lead to a reduction in the degree of hydration. A lower degree of hydration results in a more porous microstructure and uneven distribution of internal stresses, which can amplify shrinkage phenomena [41]. In fact, these salts modify the distribution of water within the cementitious matrix and influence water transport within the material’s capillary pores. Indeed, their presence can induce increased water migration by capillarity and osmosis, leading to accelerated evaporation and, consequently, increased shrinkage. Furthermore, the interaction of dissolved ions with cement hydration products has the potential to modify the material’s microstructure and sensitivity to water variations.

While experimental findings indicate an augmentation in shrinkage with industrial water utilization, a more profound examination of solute transportation within the cementitious matrix is necessary to refine these observations. In this regard, the methodology proposed by Ding et al. (2025) [42] on generalized solutions of advection–dispersion equations provides a robust theoretical framework for modeling the migration of chlorides, sulfates, and hydrocarbons in mortars. This theoretical framework could prove instrumental in analyzing the impact of these solutes on the long-term durability of mortars. A complementary study incorporating this type of modeling would provide a more nuanced understanding of the mechanisms underlying shrinkage and their correlation with the chemical composition of the water utilized.

In addition, the presence of fine particles in suspension, particularly high in collected water, can also contribute to increased shrinkage. These particles act as inclusions, which, instead of reinforcing the cementitious matrix, amplify the internal deformations of the material due to a lack of cohesion between the solid phases and the residual water. This dynamic is particularly pronounced in the case of recycled water, where industrial treatment may not completely remove fine particles and contaminants, leaving residues that continue to interfere with the formation of the cementitious matrix [43].

Finally, the difference between collected water (CW) and recycled water (RW) also amplifies the shrinkage discrepancies observed. Although recycled water is better treated, it retains a high concentration of dissolved salts, increasing internal water gradients and favoring greater deformations. Collected water, on the other hand, has a higher fine particle content and benefits from a partial filling effect, which slightly limits shrinkage compared to recycled water.

### 3.4. Mechanical Properties

The results in Table 7 show a significant reduction in the mechanical properties of mortars in both compressive strength and flexural strength as potable water (PW) is replaced by collected water (CW) or recycled water (RW). This trend is particularly marked when all potable water is replaced by RW (RW100), resulting in significantly lower performance compared to formulations using PW.

In terms of compressive strength, values after 28 days fall from 56 MPa for PW to 48 MPa for CW100 and only 35 MPa for RW100. The decrease observed with CW remains moderate, but RW causes a fall of almost 37% compared with PW. This drop can be attributed to the presence of impurities in industrial water, such as chlorides and sulfates. These chemical compounds disrupt cement hydration reactions, limiting the formation of hydrated products such as hydrated calcium silicate (C-S-H), which plays a key role in mechanical strength.

Flexural strength follows a similar trend. After 28 days, it decreases from 7.4 MPa for PW to 6.1 MPa for CW100 and 5.5 MPa for RW100. This decrease reflects a loss of cohesion in the cementitious matrix. The increase in porosity, more pronounced in formulations with RW, weakens the internal structure, compromising the mortar’s ability to resist flexural loads. A porous microstructure contains more voids, reducing the efficiency of mechanical stress transfer within the material.

The results also show that RW degrades the mechanical properties of mortars more than CW. This difference can be attributed to the chemical composition and treatment undergone by RW. Although RW undergoes a more thorough treatment process than CW, this treatment also concentrates certain impurities, notably dissolved salts such as chlorides and sulfates, which remain in the water after the solids separation and pH stabilization stages. Furthermore, recycling processes, including chemical adjustments such as carbon dioxide (CO_2_) injection, can alter the ionic composition of the water, promoting undesirable chemical interactions with cement constituents. These interactions, particularly with hydrated phases, lead to more porous and less dense microstructures, exacerbating the reduction in mechanical performance compared to CW.

In conclusion, the results highlight that the use of collected or recycled water negatively affects the mechanical performance of mortars, particularly when RW is used at 100%. These effects call for the optimization of industrial water treatment and, potentially, the addition of admixtures to compensate for the chemical and physical disturbances they cause.

## 4. Discussion

The use of industrial water, either CW or RW, in mortar formulations leads to significant changes in the mechanical and durable properties of the materials. These changes result from the complex interactions between the impurities contained in these waters and the cement constituents. While this approach is advantageous from an environmental point of view, it poses challenges in terms of controlling the final properties of mortars.

One of the primary factors influencing mortar performance is the pH of industrial water. A high pH, often observed in recycled water, can accelerate the cement hydration process by promoting the dissolution of cement particles. This leads to the rapid formation of hydration products, such as hydrated calcium silicate (C-S-H). While this acceleration can promote early strength gains, it can also upset the long-term chemical equilibrium. Too-rapid hydration can leave areas incompletely reacted, creating porous, brittle microstructures. What is more, high pH can also trigger secondary reactions, such as the alkali–silica reaction (ASR), which generates expansions and cracks in the cementitious matrix, compromising its durability.

Another key factor is the presence of sulfate ions, which react with the calcium aluminate phases of cement to form ettringite. This expansive phase induces stresses in the cement matrix, promoting cracking and structural degradation. At the same time, gypsum can also form under the effect of sulfates, exacerbating these phenomena. These mechanisms are often amplified by an increase in porosity, an effect observed in formulations containing collected or recycled water. Suspended particles present in such water disrupt the continuity of hydration products and increase the proportion of capillary pores, making the material more vulnerable to infiltration by aggressive agents and to chemical degradation.

Hydrocarbons, although often present in low concentrations, can also have significant effects on mortar properties. They interfere with adhesion between aggregates and cement paste, weakening matrix cohesion. Studies show that these substances considerably reduce mechanical strength, particularly when their concentration exceeds critical thresholds.

A major difficulty in analyzing the effects of industrial water lies in the complex interaction of these different parameters. Results depend heavily on the optimum concentrations of each substance, as well as on the chemical and physical regularity of the collected or recycled water. Variations in the composition of these waters due to changes in the industrial processes or treatments applied can lead to variable and unpredictable performance. For example, a variation in sulfate or chloride concentration can alter the chemical equilibrium and lead to unforeseen secondary reactions.

Furthermore, although the individual effects of each parameter are well documented, understanding their combined impact remains complex. Chemical and physical reactions in mortar are often synergistic, with impurities that may amplify each other or, on the contrary, neutralize each other. This interdependence makes it difficult to identify the exact contributions of each substance and, thus, to accurately predict the performance of industrial water-based mortars.

It should be noted that the injection of CO_2_ to stabilize the pH of recycled water can indirectly influence the carbonation of the mortar. This phenomenon can improve the compactness of the cementitious matrix by promoting the precipitation of calcium carbonates but can also lead to a risk of embrittlement in the long term. Further research could be carried out to assess this effect by testing the depth of carbonation.

In conclusion, although the use of industrial water represents a sustainable alternative, it requires a thorough understanding of its chemical and physical characteristics. A systematic analysis of every parameter, as well as the optimization of treatment processes, are essential to guarantee reliable, consistent mortar performance. Understanding the complex interactions between the various components of industrial water and cementitious materials remains a challenge but is crucial to maximizing environmental benefits while preserving material quality and durability. The primary challenge inherent in the gradual replacement of drinking water with industrial water pertains to the variability of its chemical composition, necessitating rigorous monitoring to ensure formulation homogeneity and to mitigate the impact on material quality.

## 5. Conclusions

This study highlighted the significant impact of using industrial water, both collected (CW) and recycled (RW), on the physico-chemical and mechanical properties of mortars. The results show that replacing potable water (PW) with industrial water leads to significant changes in porosity, density, shrinkage, and compressive and flexural strengths.

Firstly, the increase in porosity and decrease in density observed for formulations containing CW and RW indicate a deterioration in the compactness of the cementitious matrix. This is mainly attributed to the presence of impurities such as fine particles and dissolved salts (chlorides, sulfates) in these waters. These impurities disrupt hydration reactions, limiting the formation of essential products such as hydrated calcium silicate (C-S-H) and increasing the proportion of capillary pores in the material.

Secondly, the increase in delayed shrinkage observed in formulations with RW and CW highlights a weakening in the dimensional stability of mortars. This phenomenon is exacerbated by the high concentration of dissolved salts in these waters, which promote the evaporation of residual water and increase the mortars’ susceptibility to shrinkage.

Mechanical properties, notably compressive and flexural strengths, were also significantly impacted. Formulations with RW show a more pronounced loss of performance than those with CW, with compressive and flexural strengths reduced by 37% and 26%, respectively, compared to PW. These reductions are directly linked to disturbances in hydration reactions and increased porosity, aggravated in the case of RW by chemical treatments (such as pH adjustment) that concentrate impurities.

Recycled water (RW) was found to have a more negative effect than collected water (CW) on all the properties studied. This underlines the importance of better control of recycled water treatment processes to avoid the accumulation of undesirable compounds while reducing the risk of harmful chemical interactions with cement. Conversely, collected water, although less treated, showed a less severe impact, suggesting that simple adjustments to its treatment could enable wider use with controlled effects on mortars.

In analyzing the effects of industrial water, a particular difficulty concerns the complex interaction of different parameters. Results depend heavily on the concentration of each substance, as well as on the chemical and physical consistency of the collected or recycled water. Variations in the composition of these waters due to changes in the industrial processes or treatments applied can lead to variable and unpredictable performances that are difficult to analyze.

In conclusion, although the use of industrial water is an interesting solution for reducing drinking water consumption in the concrete industry, it requires significant precautions to limit its adverse effects on mortar properties. The results of this study highlight the need to optimize industrial water treatment processes, as well as to explore complementary solutions, such as the addition of admixtures or the reformulation of cementitious mixes, to offset the negative impacts observed. These prospects pave the way for the sustainable, controlled use of industrial water in construction while maintaining optimum mechanical and durable performance.

## Figures and Tables

**Table 1:** Cement characteristics as given in the technical sheet.

Data	CEM II/A-LL 52.5 R
SO_3_	2.5%
MgO	0.7%
Na_2_O	0.2%
Cl^−^	0.02%
Blaine fineness	3700–4000 cm^2^/g
Specific density	3030 kg/m^3^
Compressive strength at 1 day	21–28 MPa
Compressive strength at 2 days	34–42 MPa
Compressive strength at 28 days	56–66 MPa

**Table 2:** Sand physical properties as given in the technical sheet.

Physical Properties	0/4 Sand
Density (kg/m^3^)	2630
Water absorption (%)	0.1
Sand equivalent	81.3
Fineness modulus	2.3

**Table 3:** Water analysis.

Parameter	Potable Water	Collected Water	Recycled Water
Suspended Matter	Absence	3400 mg/L	93 mg/L
pH	7.1 at 20 °C	10.9 at 20 °C	7.5 at 20 °C
Chlorides (Cl^−^)	41 mg/L	80 mg/L	50 mg/L
Sulfates (SO_4_^2−^)	37 mg/L	90 mg/L	60 mg/L
Na_2_O	17 mg/L	25 mg/L	20 mg/L
K_2_O	4.5 mg/L	10 mg/L	6.5 mg/L
Phosphates (PO_4_^3−^)	<0.037 mg/L	0.05 mg/L	0.04 mg/L
Nitrates (NO_3^−^_)	32 mg/L	60 mg/L	40 mg/L
Total Sugars	60 mg/L	200 mg/L	120 mg/L
Lead (Pb^2+^)	0.001 mg/L	0.02 mg/L	0.01 mg/L
Zinc (Zn^2+^)	0.12 mg/L	0.3 mg/L	0.2 mg/L
Dissolved Salts	662 mg/L	1500 mg/L	900 mg/L
Chromium VI (Cr^6+^)	<0.01 mg/L	0.02 mg/L	<0.01 mg/L
Hydrocarbon Index	<0.1 mg/L	5 mg/L	0.5 mg/L

**Table 4:** Mortar formulations.

Component	Cement (kg/m^3^)	Sand (kg/m^3^)	Potable Water (kg/m^3^)	Collected Water (kg/m^3^)	Recycled Water (kg/m^3^)
Ref	450	1350	225	0	0
CW25	450	1350	168.75	56.25	
CW50	450	1350	112.5	112.5	
CW75	450	1350	56.25	168.75	
CW100	450	1350	0	225	
RW25	450	1350	168.75		56.25
RW50	450	1350	112.5		112.5
RW75	450	1350	56.25		168.75
RW100	450	1350	0		225

**Table 5:** Mortar physical properties.

Formulation	Porosity (%)	Density (kg/m^3^)
Ref	19	2010
CW25	20	2008
CW50	21	2006
CW75	22	2001
CW100	24	1996
RW25	20	2006
RW50	24	2004
RW75	22	1995
RW100	24	1990

**Table 6:** Mortar total shrinkage.

Formulation	Total Shrinkage (µm/m)		
	1 Day	3 Days	28 Days
Ref	500	610	715
CW25	510	615	720
CW50	550	620	730
CW75	560	625	735
CW100	562	633	740
RW25	515	617	720
RW50	575	630	722
RW75	600	635	740
RW100	610	640	815

**Table 7:** Mechanical properties of mortars.

Formulation	Compressive Strength (MPa)		Flexural Strength (MPa)	
	14 Days	28 Days	14 Days	28 Days
Ref	50	56	7.1	7.4
CW25	47	51	6.4	6.6
CW50	46	50	6.3	6.5
CW75	44	48	6.2	6.4
CW100	38	47	5.1	6.1
RW25	44	46	6.5	7
RW50	42	48	6.1	6.2
RW75	40	44	5.9	6
RW100	32	35	4.9	5.5

## Data Availability

The data presented in this study are available on request from the corresponding author due to privacy.
